# Supplementary material for: Mechanodetection of neighbor plants elicits adaptive leaf movements through calcium dynamics
Source: Nat Commun. 2023 Sep 20;14:5827. doi: 10.1038/s41467-023-41530-0 (PMC10511701; doi:10.1038/s41467-023-41530-0)
Supplement: Supplementary file 1 — Supplementary Information [file 41467_2023_41530_MOESM1_ESM.pdf]

Supplementary information for:

Mechanodetection of neighbor plants elicits adaptive leaf movements through calcium dynamics

Chrysoula K. Pantazopoulou[1*], Sara Buti[1], Chi Tam Nguyen[2], Lisa Oskam[1], Daan A. Weits[1]

Edward E. Farmer[2], Kaisa Kajala[1] and Ronald Pierik[1,3*]

[1]*Plant-Environment Signaling, Institute of Environment Biology, Utrecht University, The Netherlands*

[2]*Department of Plant Molecular Biology, University of Lausanne, Switzerland*

[3]*Laboratory of Molecular Biology, Wageningen University, The Netherlands*

*Corresponding authors: c.pantazopoulou@uu.nl and ronald.pierik@wur.nl

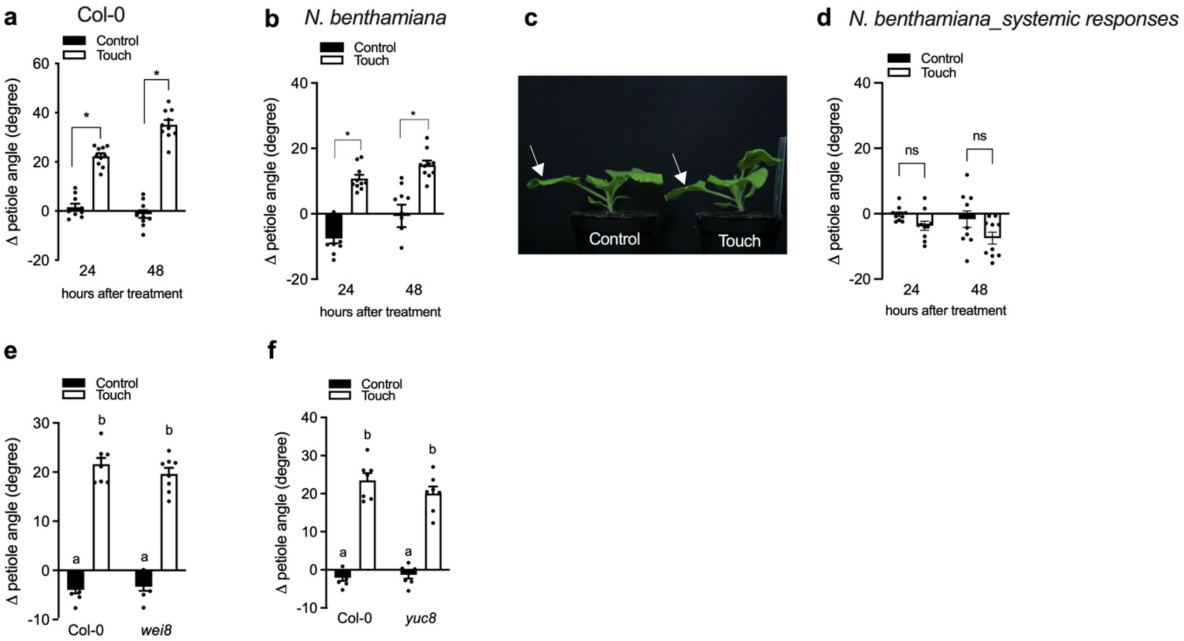

Supplementary Figure 1: Touch-induced hyponasty in *A. thaliana* and *N. benthamiana* wild types, as well as in *A. thaliana* auxin biosynthesis mutants. The differential petiole angle of touched leaves in (**a**) *A. thaliana* Col-0 (n-10) and (**b**) *N. benthamiana* (n=10) after 24 h and 48 h. (**c**) Representative photo of (**b**) *N. benthamiana* leaves touching transparent tags after 24 h. (**d**) The differential petiole angle of systemic leaves (white arrow) in touch-treated *N. benthamiana*. The differential petiole angle of touch-induced hyponasty in Col-0 compared to (**e**) *wei8* and (**f**) *yuc8* mutants 24h after touch treatment. (**a**,**b**,**d**) n=10 biologically independent replicates, (**e**) n=8 biologically independent replicates and (**f**) n=7 biologically independent replicates. Touch was induced by a gently positioned transparent tag next to the leaf. Black dots represent the individual data. Data represent mean ± SE. Statistically significant differences are indicated with asterisk, unpaired t test or different letters (two-way ANOVA with Tukey's post hoc test ($p < 0.05$).

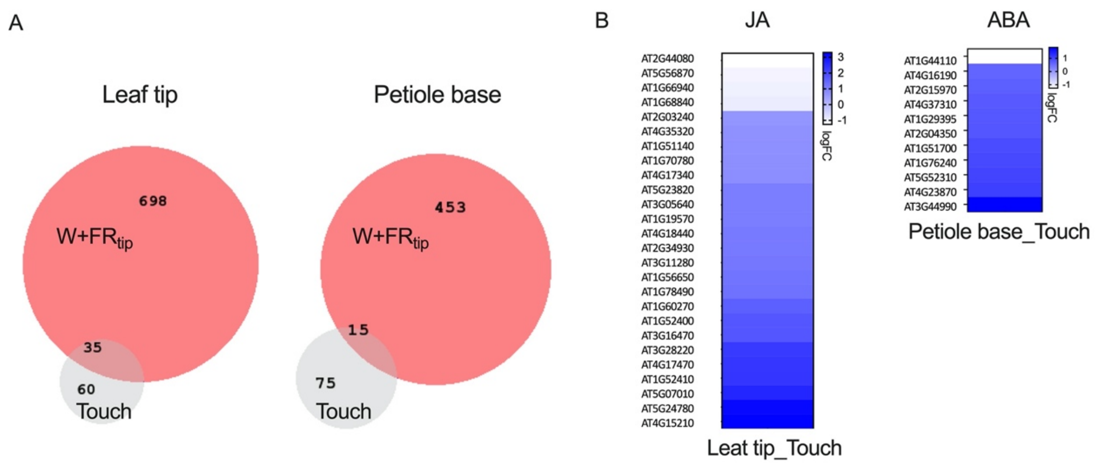

Supplementary Figure 2: Different molecular pathways are activated during touch- and FR-induced hyponasty. (**a**) Venn diagrams illustrate the differentially expressed genes (DEGs) common to the touch (white circle) and local-FR treatment (red circle) in the leaf tip (the first Venn diagram) and petiole base (the second Venn diagram). (**b**) Heatmap representation of the change in expression level of DEGs that are associated with JA and ABA in the leaf tip ("Leaf tip_Touch") and petiole base ("Petiole base_Touch") respectively in response to touch. Blue color represents the upregulated genes while the white represents the downregulated genes

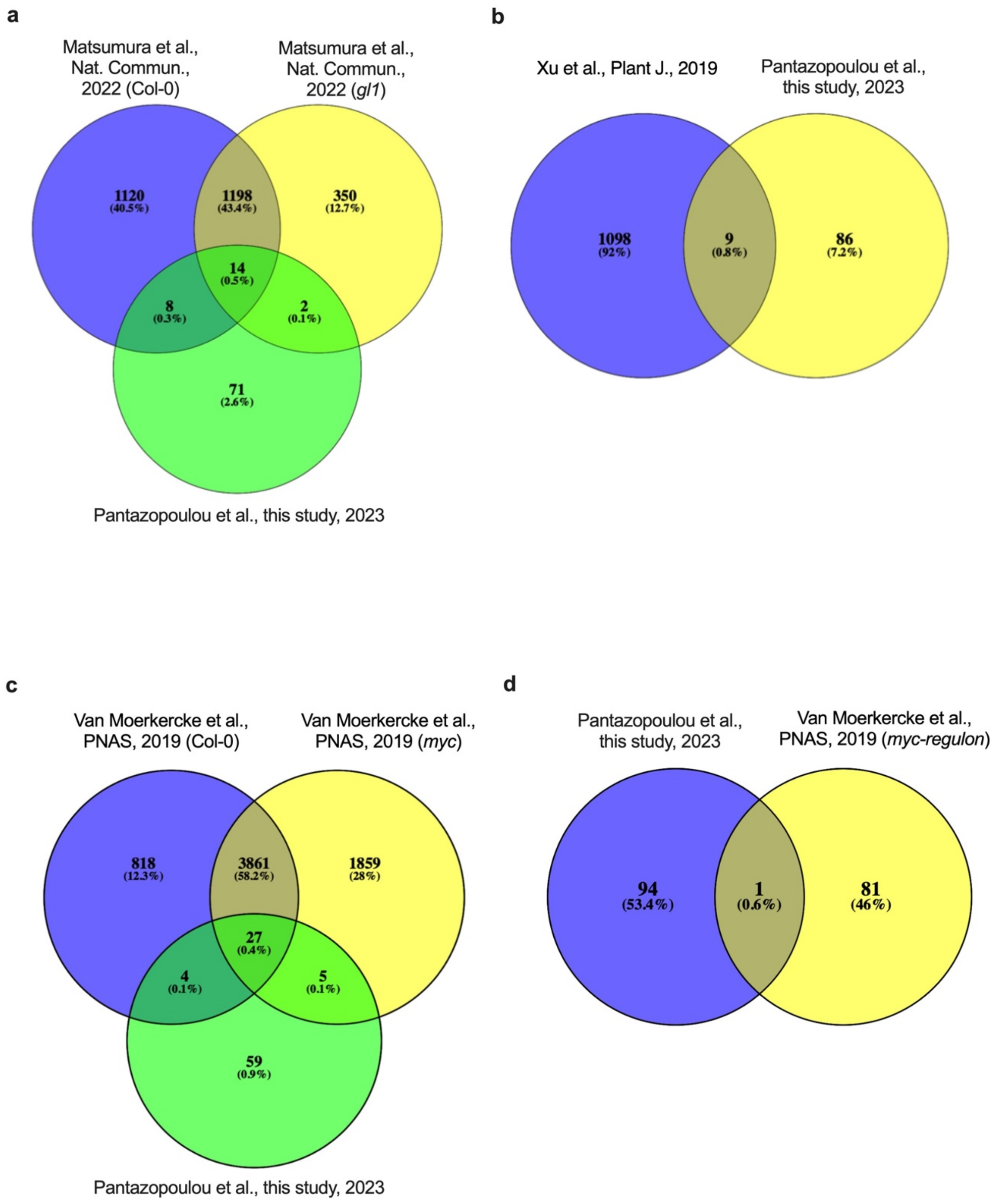

Supplementary Figure 3: Comparison of previously published transcriptome datasets against those collected during touch-induced hyponasty. (**a**) The common DEGs between brushing treatment from any time point of Col-0 and *gl1* (Matsumura *et al*., 2022) and touch treatment at the leaf tip in the current study (Fisher exact test for the comparison between *gl1* (brushing) and Col-0 leaf tip (touch) is p=0.0015, while the Fisher exact test for the

comparison between Col-0 (brushing) and Col-0 leaf tip (touch) is p<0.00001). (**b**) The common DEGs between brushing treatment at 30 min of Col-0 (Xu *et al*., 2019) and touch treatment at the leaf tip in this study. The Fisher exact test for the comparison between Col-0 (30 min time point after brushing) and Col-0 leaf tip (touch) is p=0.2625). (**c**) The common DEGs between water spray treatment of Col-0 and *myc2myc3myc4* (Van Moerkercke *et al.*, 2019) and touch treatment at the leaf tip in this study. The Fisher exact test for the comparison between *MYC234*-independent response (*myc2myc3myc4* water spray) and col-0 leaf tip (touch) is p=0.0005, while the Fisher exact test statistic value for the comparison between *MYC234*-dependent response (Col-0 unique (i.e. not shared with *myc2myc3myc4*) water spray) and Col-0 leaf tip (touch) is p=0.0225). (**d**) The overlap between DEGs of touch treatment at the leaf tip (this study) and the MYC2-regulon (Van Moerkercke *et al*., 2019). The Fisher exact test for this comparison is p=0.2106. Venn diagrams were made by Venny (https://bioinfogp.cnb.csic.es/tools/venny/). Gene IDs per section of each of the VENN diagrams are provided in supplementary Data 2.

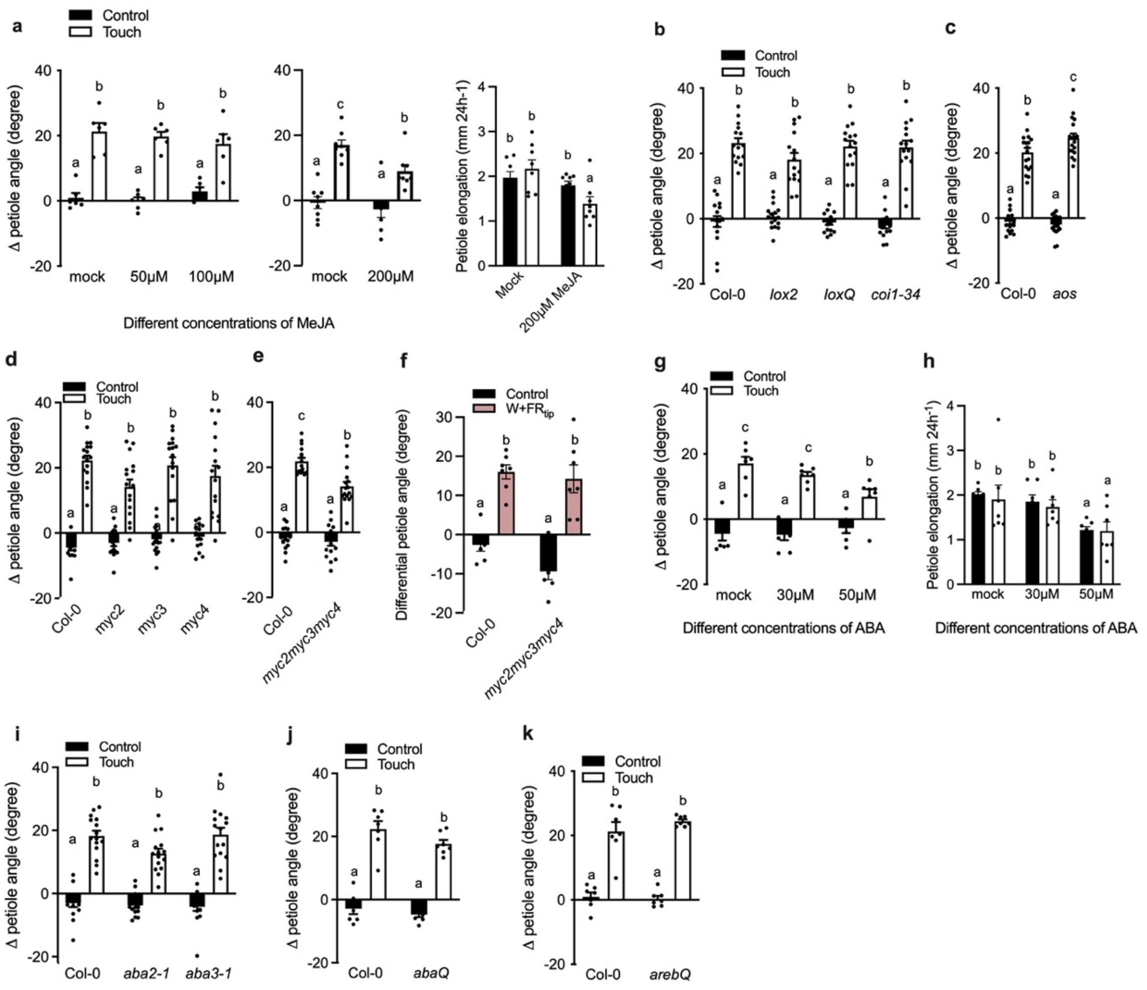

Supplementary Figure 4: Touch-induced hyponasty is not specifically regulated by ABA or JA. Differential petiole angle or elongation after exogenous application of different (**a**) MeJA (50 µM, 100 µM and 200 µM) concentrations after 24 h of touch treatment. Differential petiole angle of Col-0 compared to (**b**) *lox2*, *loxQ*, *Coi-34*, (**c**) *aos* (**d**) *myc2*, *myc3*, *myc4*, and (**e**) *myc2myc3myc4* mutants after 24 h of touch treatment. (**f**) Differential petiole angle of Col-0 compared to *myc2myc3myc4* upon local FR (“W+FR_{tip}”) treatment. (**g**) Differential petiole angle and (**h**) petiole elongation after exogenous application of different ABA concentrations (30µM and 50µM) after 24h of touch treatment. Differential petiole angle of Col-0 compared to (**i**) *aba2-1* and *aba3-1*, (**j**) *abaQ* and (**k**) *arebQ* mutants after 24 h of touch treatment. (**a**)

n=6 biologically independent replicates for concentrations 50µM and 100 µM. n=8 biologically independent replicates for concentration 200µM. (**b,i**) n=15 biologically independent replicates. (**c**) n=18 biologically independent replicates. (**d**,**e**) n=16 biologically independent replicates. (**f**,**g**,**h**,**j**,**k**) n=7 biologically independent replicates. Touch was induced by a gently positioned transparent tag next to the leaf. Black dots represent the individual data. Data represent mean ± SE. Different letters indicate significant differences (two-way ANOVA with Tukey's post hoc test; $p < 0.05$).

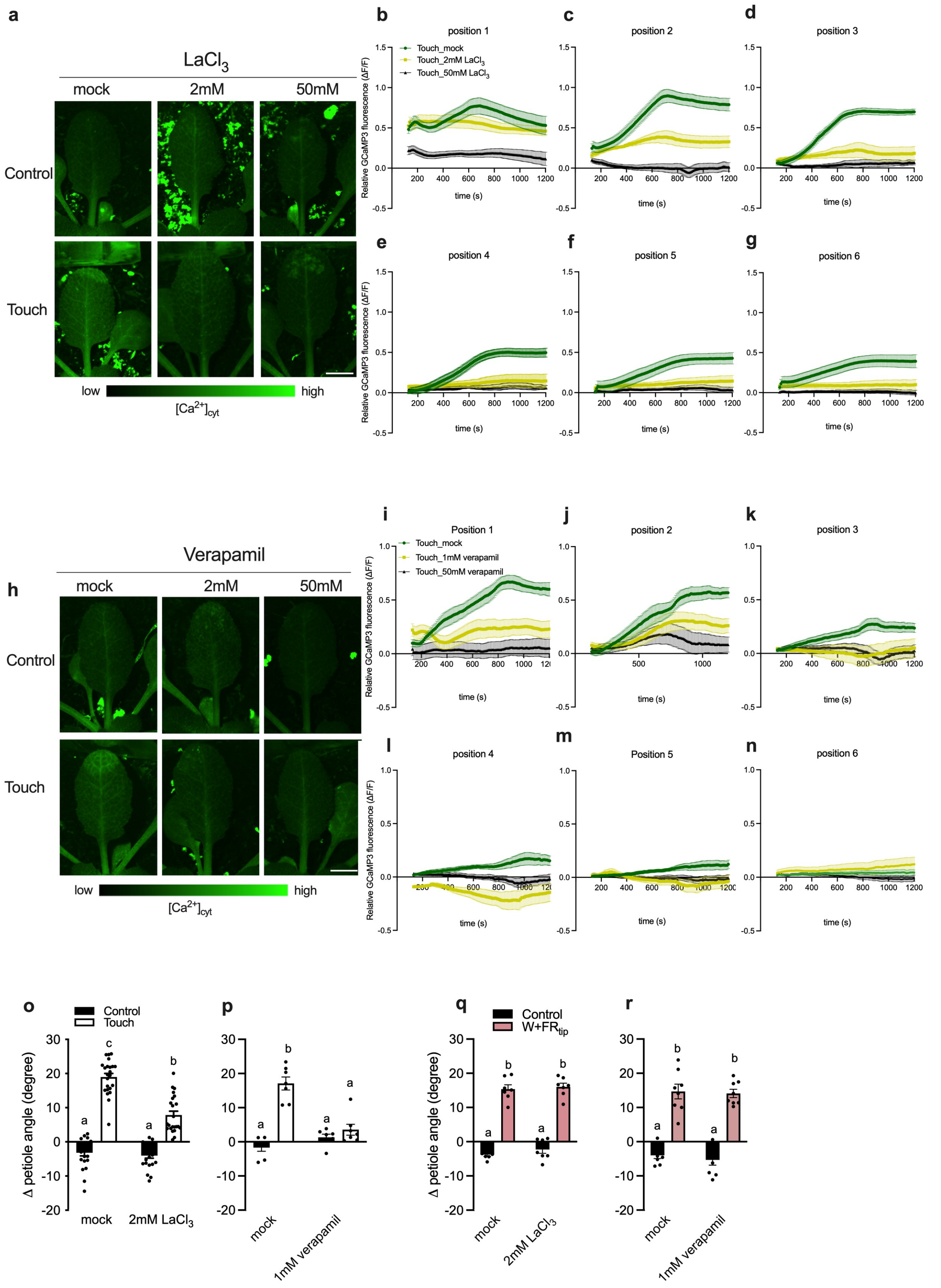

Supplementary Figure 5: Inhibition of $[Ca^{2+}]_{cyt}$ at leaf the tip where touch is applied markedly diminishes calcium wave across leaf. Two Ca^{2+} inhibitors ($LaCl_3$: **a-g**) and verapamil (**h-n**) both

reduce touch-induced calcium waves. Six different positions were used to measure the GCaMP3 fluorescence in control or touch treatment after exogenous application of mock or 2 mM or 50 mM $LaCl_3$ at the leaf tip. Time course data express fluorescence during touch relative to control (no touch) for each treatment. Relative GCaMP3 fluorescence (ΔF/F touch treatment$_{tx}$ - ΔF/F control$_{averagetx}$) GCaMP3 fluorescence in leaf tip (position 1), primary vein of the lamina (position 2 and 3), lamina-petiole junction (position 4), in the middle of the adaxial site of the petiole (position 5) and in the adaxial site of the petiole base (position 6) upon mock (green line), 2 mM LaCl3 or 1 mM verapamil (yellow line) and 50 mM $LaCl_3$ or verapamil (black line) treatment during touch treatment. Touch treatment (a-n) started at t=2 min (120 sec) and fluorescence was followed for 20 minutes. Differential petiole angle of Col-0 after 24h of (**o,p**) touch ("Touch";n=23 (o), n=7 (p)) or (**q,r**) local FR ("W+FR$_{tip}$" ; n=7 (q), n=8 (r)) treatment after exogenous application at the whole leaf of mock or 2 mM $LaCl_3$ or 1mM verapamil. (**a-g**) n=10 and (**h-n**) n=8 biologically independent replicates. (**o**) n=23, (**p,q**) n=7 and (**r**) n=8 biologically independent replicates. Touch was induced by a gently positioned transparent tag next to the leaf. Black dots represent the individual data. Different letters indicate significant differences (two-way ANOVA with Tukey's post hoc test; $p < 0.05$). Data represent mean $\pm$ SE.

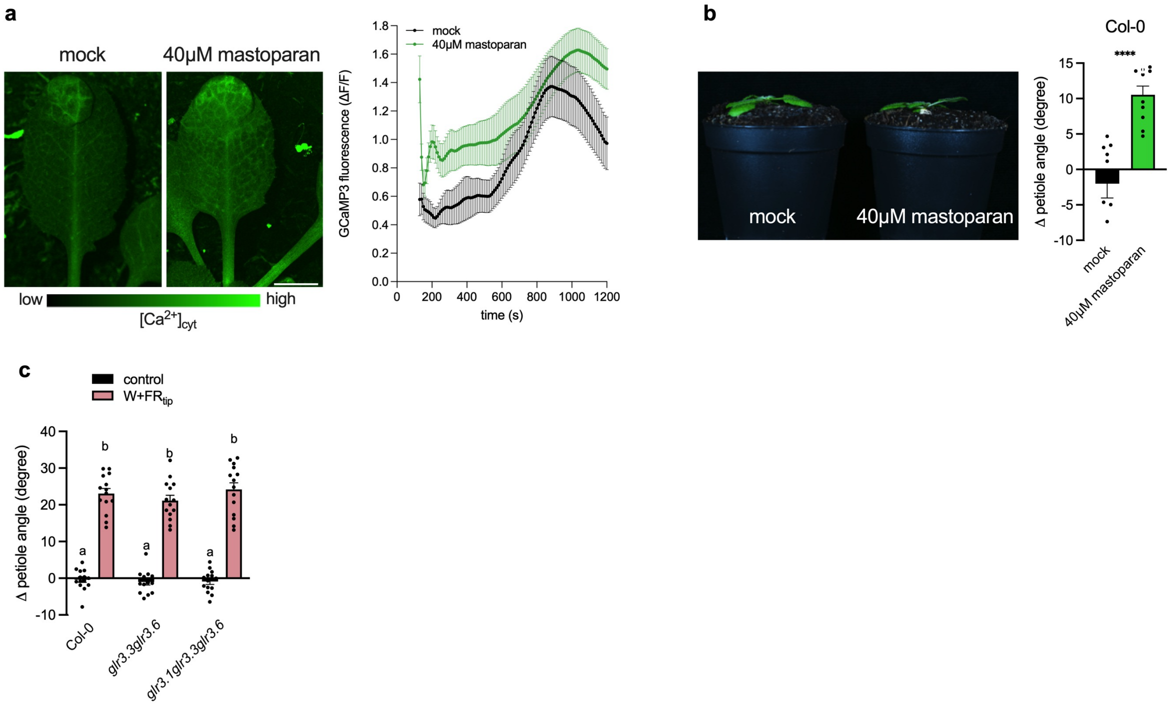

Supplementary Figure 6: The *glr* mutants wild-type-like hyponasty response to local FR treatment, while Mastoparan is able to induce hyponastic response without touch. (**a**) GCaMP3 fluorescence in leaf tip upon 10 μl of exogenous droplet application of mock or 40 μM Mastoparan in the leaf tip using the fluorescent cytosolic calcium biosensor *UBQ10p::GCaMP3*. Relative GCaMP3 fluorescence (ΔF/F touch treatment$_{tx}$ - ΔF/F control$_{averagetx}$) of GCaMP3 fluorescence in leaf tip (n=8). (**b**) Differential petiole angle of Col-0 after 4 μl of exogenous application of mock or 40 μM Mastoparan in the leaf tip, middle of the leaf and petiole-lamina junction (n=10). (**c**) Differential petiole angle of Col-0 compared to *glr3.3aglr3.6a* and *glr3.1glr3.3glr3.6,* after 24 h of local FR treatment (W+FR$_{tip}$), n=14.

(**a**) n=8, (**b**) n=10 and (**c**) n=14 biologically independent replicates. Touch was induced by a gently positioned transparent tag next to the leaf. Black dots represent the individual data. Data represent mean ± SE. Statistically significant differences are indicated with asterisk, unpaired t test or different letters (two-way ANOVA with Tukey's post hoc test ($p < 0.05$).

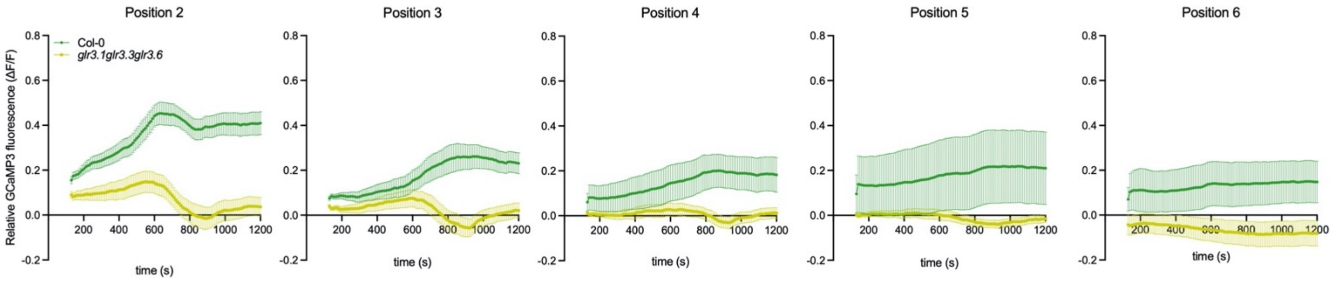

Supplementary Figure 7: GLRs decrease calcium signal propagation away from the leaf tip upon touch stimulation. Relative GCaMP3 fluorescence (ΔF/F touch treatment$_{tx}$ - ΔF/F control$_{averagetx}$) of GCaMP3 fluorescence in primary vein of the lamina (position 2 and 3), lamina-petiole junction (position 4), in the middle of the adaxial site of the petiole (position 5) and in the adaxial site of the petiole base (position 6) in Col-0 (green line), and *glr3.1glr3.3glr3.6* (yellow line) during touch treatment. For leaf tip (position 1), see Fig. 4d. Data were normalized to control treatments. Touch treatment started at t=2 min (120 sec) and fluorescence was followed for 20 minutes. n=12 biologically independent replicates for *UBQ10pro::GCaMP3* and n=15 biologically independent replicates for *glr3.1glr3.3glr3.6* with *UBQ10pro::GCaMP3.* Touch was induced by a gently positioned transparent tag next to the leaf. For this experiment we used the fluorescent cytosolic calcium biosensor *UBQ10p::GCaMP3* and *glr3.1glr3.3glr3.6* with *UBQ10p::GCaMP3*. Data represent mean $\pm$ SE.

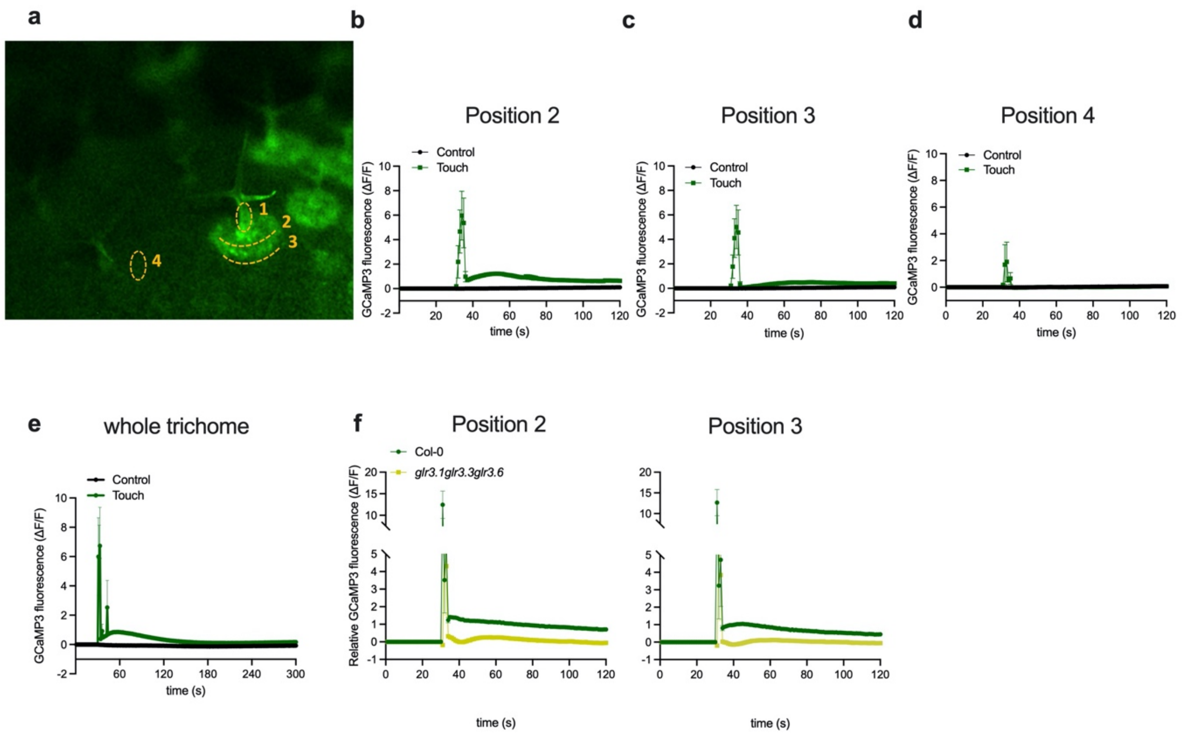

Supplementary Figure 8: Cytosolic calcium levels in the trichomes increase upon mechanostimulation. (**a**) Four different positions were used to measure the GCaMP3 fluorescence within the trichome upon control or touch treatment, using the fluorescent cytosolic calcium biosensor *UBQ10p::GCaMP3*. (**b**-**d**) Time course of GCaMP3 fluorescence in skirt cells (position 2), surrounding trichome cells (position 3) and close to a neighbor trichome (position 4) upon control (black line) and touch (green line) treatment. For position 1, see Fig. 5b. Touch treatment started at t=30 sec and fluorescence was followed for 2 minutes. (**e**) Time course of GCaMP3 fluorescence in the whole trichome upon control (black line) and touch (green line) treatment. Touch treatment started at t=30 sec and fluorescence was followed for 5 minutes. (**f**) Relative GCaMP3 fluorescence (ΔF/F touch treatment$_{tx}$ - ΔF/F control$_{averagetx}$) of GCaMP3 fluorescence in skirt cells (position 2) and surrounding trichome cells (position 3) upon touch treatment in Col-0 (green line) and *glr3.1glr3.3glr3.6* (yellow line). For this experiment we used the fluorescent cytosolic calcium biosensor

UBQ10p::GCaMP3 and *glr3.1glr3.3glr3.6* with *UBQ10p::GCaMP3*. For position 1, see Fig. 5e. Touch treatment started at t=30 sec and fluorescence was followed for 2 minutes. To induce Touch, trichomes were gently touched with a toothpick. (**b-d**) For "Control" treatment n=11 while for "Touch" treatment n=10 biologically independent replicates. (**e**) For "Control" treatment n=18 while for "Touch" treatment n=21 biologically independent replicates. (**f**) n=12 biologically independent replicates. Data represent mean $\pm$ SE.

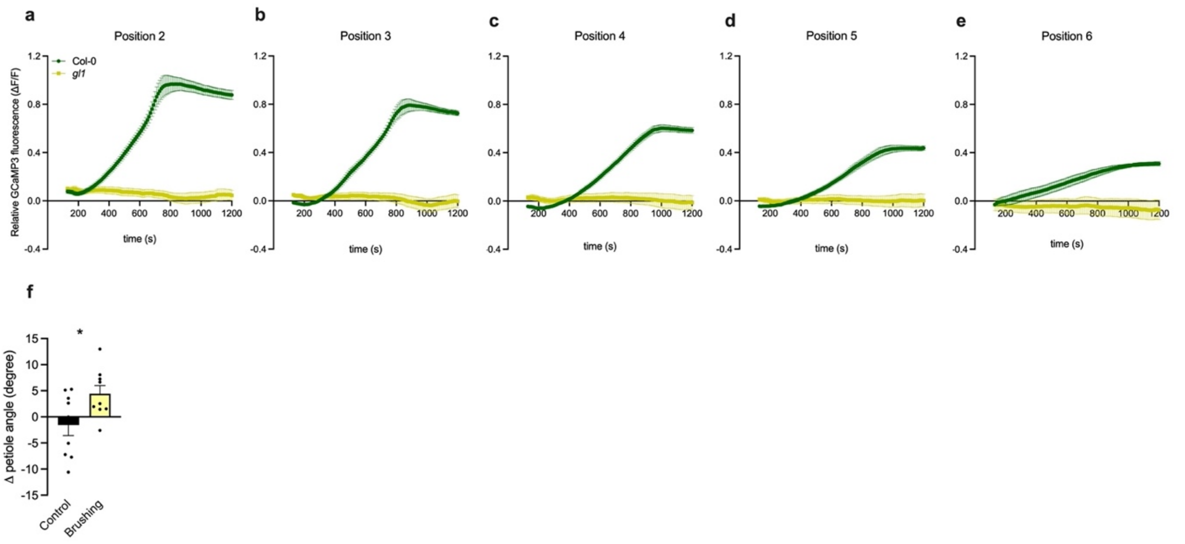

Supplementary Figure 9: Calcium induction and transport are trichome dependent and trichome brushing can promote mild hyponasty. (**a-e**) Relative GCaMP3 fluorescence (ΔF/F touch treatment$_{tx}$ - ΔF/F control$_{averagetx}$) of GCaMP3 fluorescence in primary vein of the lamina (position 2 and 3), lamina-petiole junction (position 4), in the middle of the adaxial site of the petiole (position 5) and in the adaxial site of the petiole base (position 6) in Col-0 (green line), and *gl1* (yellow line) during touch treatment. For leaf tip (position 1), see Fig. 6b. (**a-e**) n=14 biologically independent replicates. Touch was induced by a gently positioned transparent tag next to the leaf. For this experiment we used the fluorescent cytosolic calcium biosensor *UBQ10p::GCaMP3* and *gl1* with *UBQ10p::GCaMP3*. (**f**) Repeated mild brushing of Col-0 leaf tip trichomes every 10 minutes during the photoperiod, starting at ZT = 2 promotes petiole angles after 7 hrs of treatment, * indicates significant difference. n=8 biologically independent replicates. Black dots represent the individual data. Data represent mean $\pm$ SE.

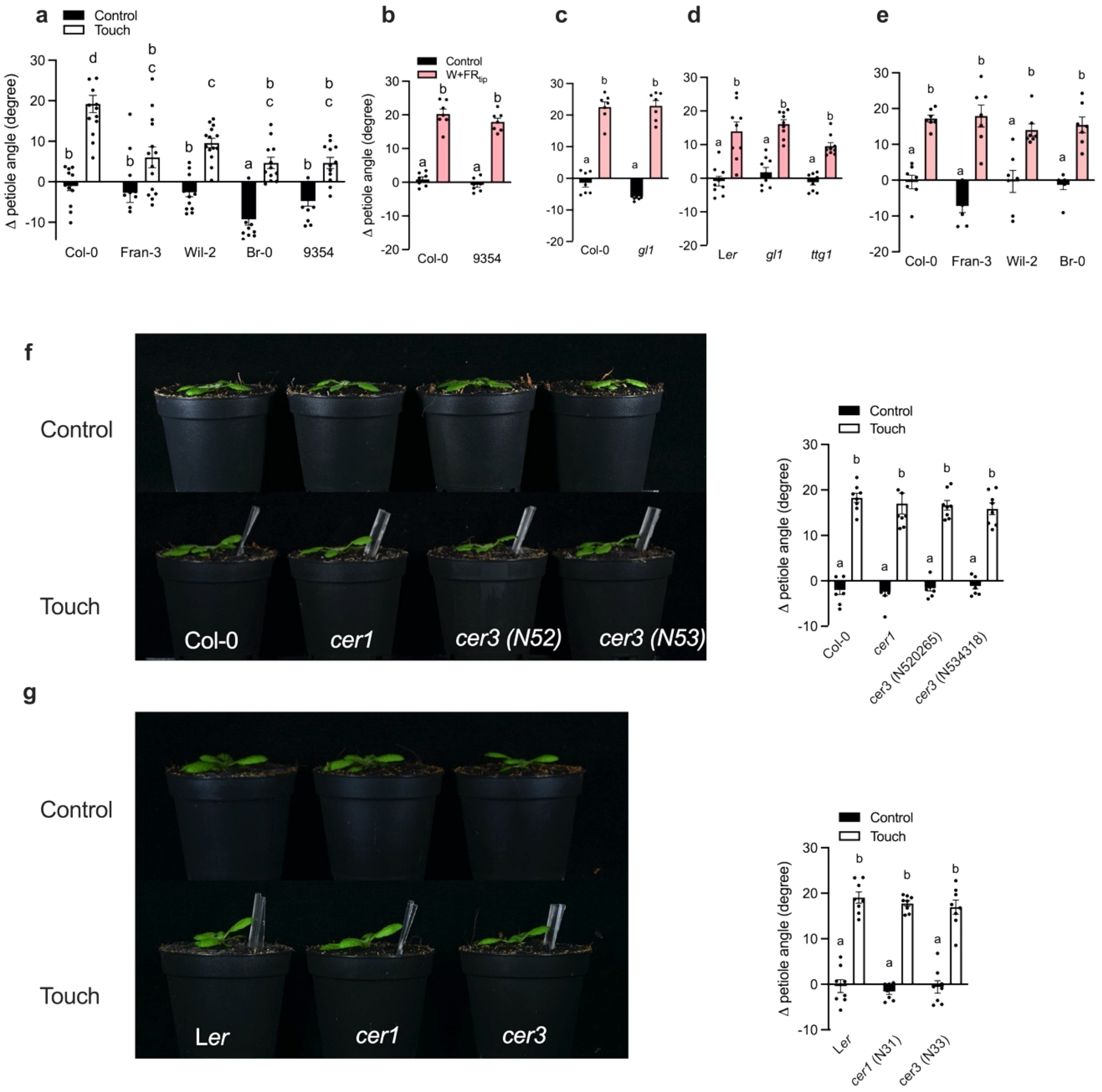

Supplementary Figure 10: Trichomes play a key role in the touch-induced hyponasty but not in the local FR-induced hyponasty and cuticle mutants have undisturbed touch-induced hyponasty. (**a**) Differential petiole angle of Arabidopsis accessions without trichomes (Fran-3, Wil-2, Br-0, 9354) compared to an accession with trichomes (Col-0) after 24 h of touch treatment. (**b-e**) Differential petiole angle of (**b**) 9354, (**c**) *gl1* (Col-0 background), (**d**) *gl1* and *ttg1* (L*er* background) and (**e**) Arabidopsis accessions without trichomes (Fran-3, Wil-2, Br-0, 9354) compared to their wild types or accession*,* after 24 h of local FR treatment (W+FR_{tip}).

(**f,g**) Leaf angles responses to touch of (**f**) *cer1*, *cer3* (520265), *cer3* (N534318) and (**g**) *cer1* (N31) and *cer3* (N33) compared to their wild types. Black dots represent the individual data. (**a**) n=12, (**b**,**c**,**e**) n=7, (**d**,**f**,**g**) n=8 biologically independent replicates. Data represent mean ± SE. Touch was induced by a gently positioned transparent tag next to the leaf. Different letters indicate significant differences (two-way ANOVA with Tukey's post hoc test; $p < 0.05$).

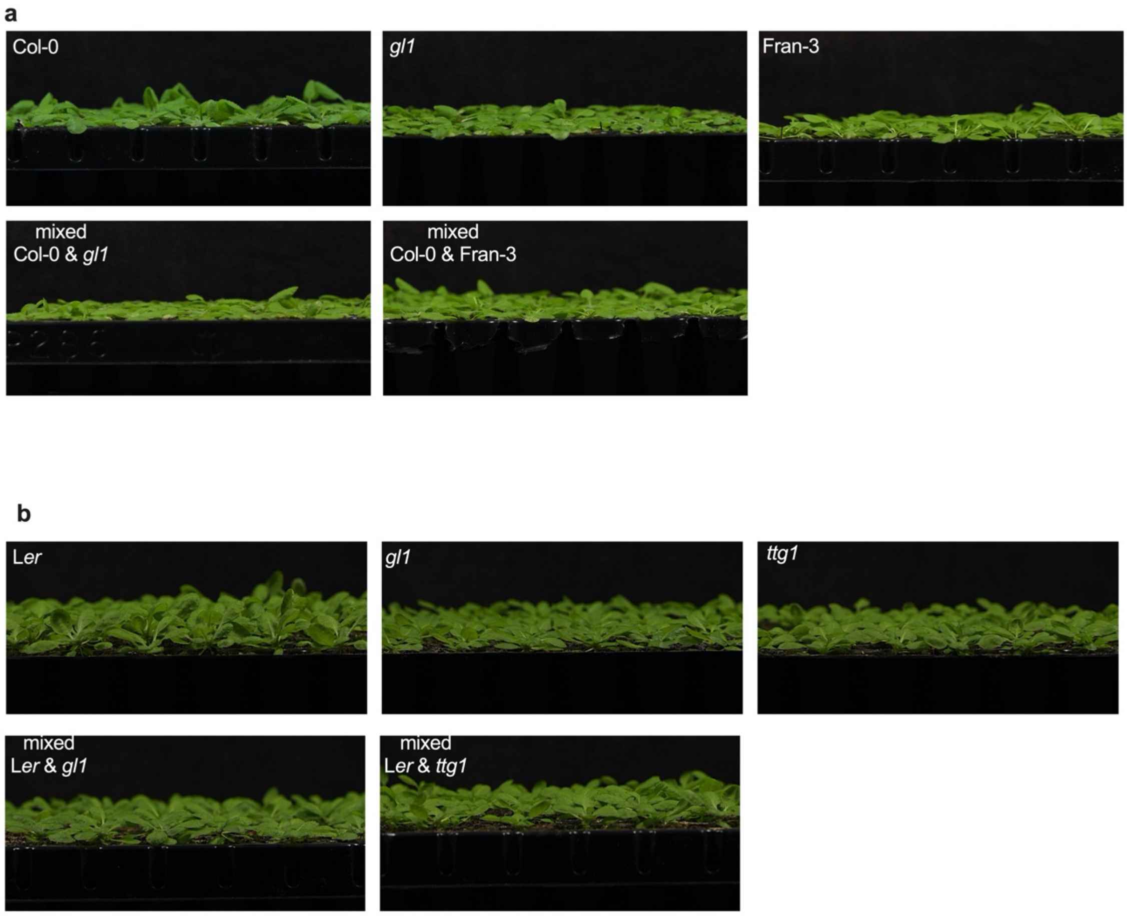

Supplementary Figure 11: Pictures illustrating the monoculture canopy of (**a**) Col-0, *gl1* and Fran-3 and their mixtures with Col-0, (**b**) L*er*, *gl1* and *ttg1* and and their mixtures with L*er*.

a

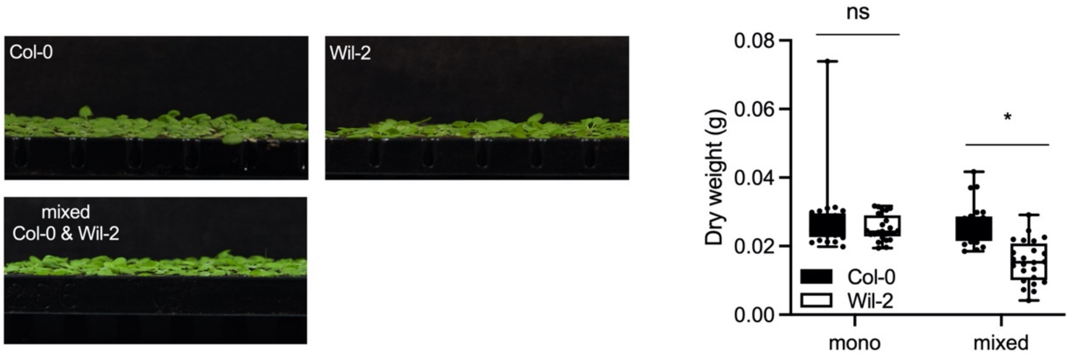
Col-0
Wil-2
mixed
Col-0 & Wil-2
ns
*
Dry weight (g)
0.08
0.06
0.04
0.02
0.00
Col-0
Wil-2
mono
mixed

b

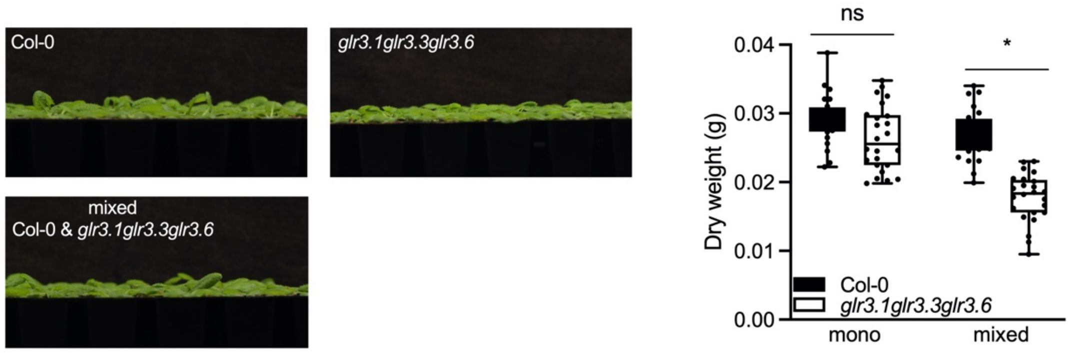
Col-0
glr3.1glr3.3glr3.6
mixed
Col-0 & glr3.1glr3.3glr3.6
ns
*
Dry weight (g)
0.04
0.03
0.02
0.01
0.00
Col-0
glr3.1glr3.3glr3.6
mono
mixed

c

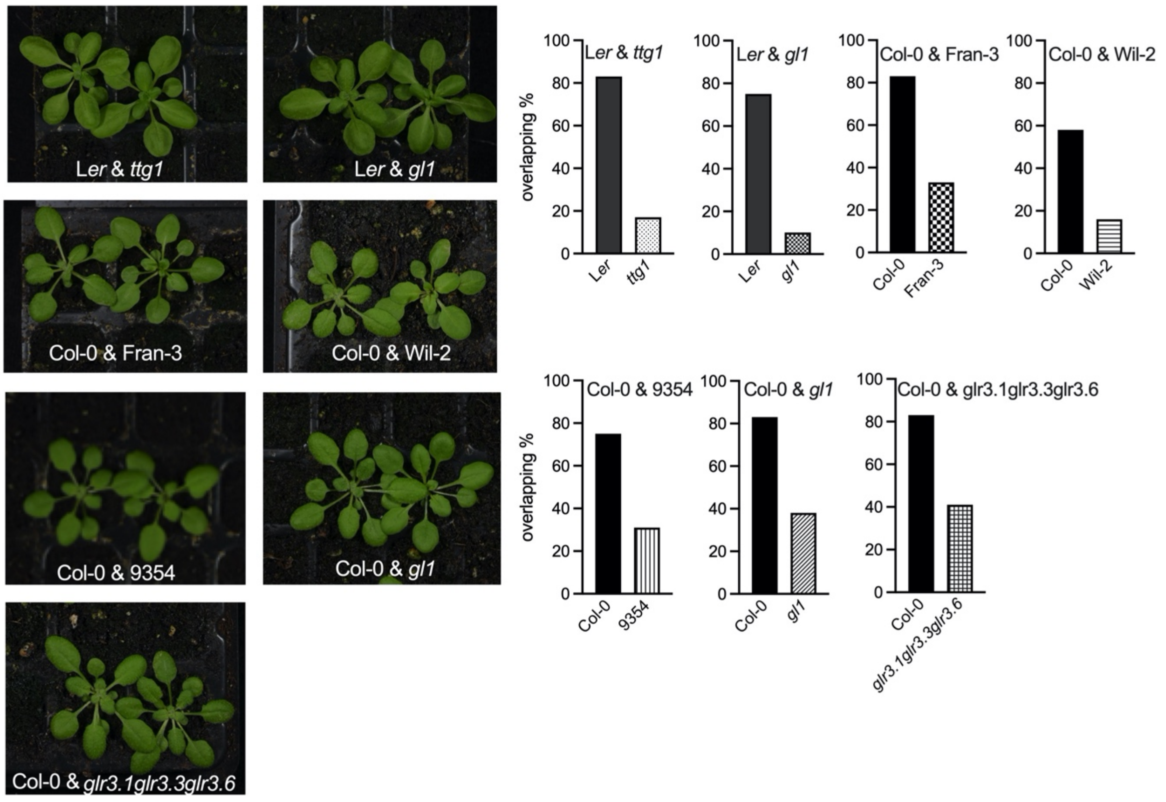
Ler & ttg1
Ler & gl1
Col-0 & Fran-3
Col-0 & Wil-2
Col-0 & 9354
Col-0 & gl1
Col-0 & glr3.1glr3.3glr3.6
overlapping %
100
80
60
40
20
0
Ler
ttg1
gl1
Col-0
Fran-3
Wil-2
9354
Col-0 & glr3.1glr3.3glr3.6
glr3.1glr3.3glr3.6

Supplementary Figure 12: Competition and paired-individual assays for different accessions and genotypes. (**a**) Pictures illustrating the monoculture canopy of Col-0 and Wil-2 and the mixed canopy of both accessions together. Dry weight of Col-0 and Wil-2 in monoculture and mixed canopies (together Col-0 and Wil-2). (**b**) Pictures illustrating the monoculture canopy of Col-0 and *glr3.1glr3.3glr3.6* and the mixed canopy of both genotypes together. Dry weight of Col-0 and *glr3.1glr3.3glr3.6* in monoculture and mixed canopies (together Col-0 and *glr3.1glr3.3glr3.6*). (**c**) Overlapping experiment of individual pairs of 28 days old plants growing next to each other (distance 2 cm). The overlap experiment was performed with the following combinations L*er* & ttg1, L*er* & gl1, L*er* & Fran-3, L*er* & Wil-2, Col-0 & 9354, Col-0 and *gl1* and Col-0 & *glr3.1glr3.3glr3.6*. Black dots represent the individual data. (**a**,**b**) n=24 and (**c**) n=12 biologically independent replicates. The boxes show the inter-quartile ranges and the whiskers show minimum and maximum values but also plot the individual values as dots. The line at the middle of the box represents the median. Data represent mean ± SE. Different letters or asterisks indicate significant differences (two-way ANOVA with Tukey's post hoc test or t-test; $p < 0.05$).

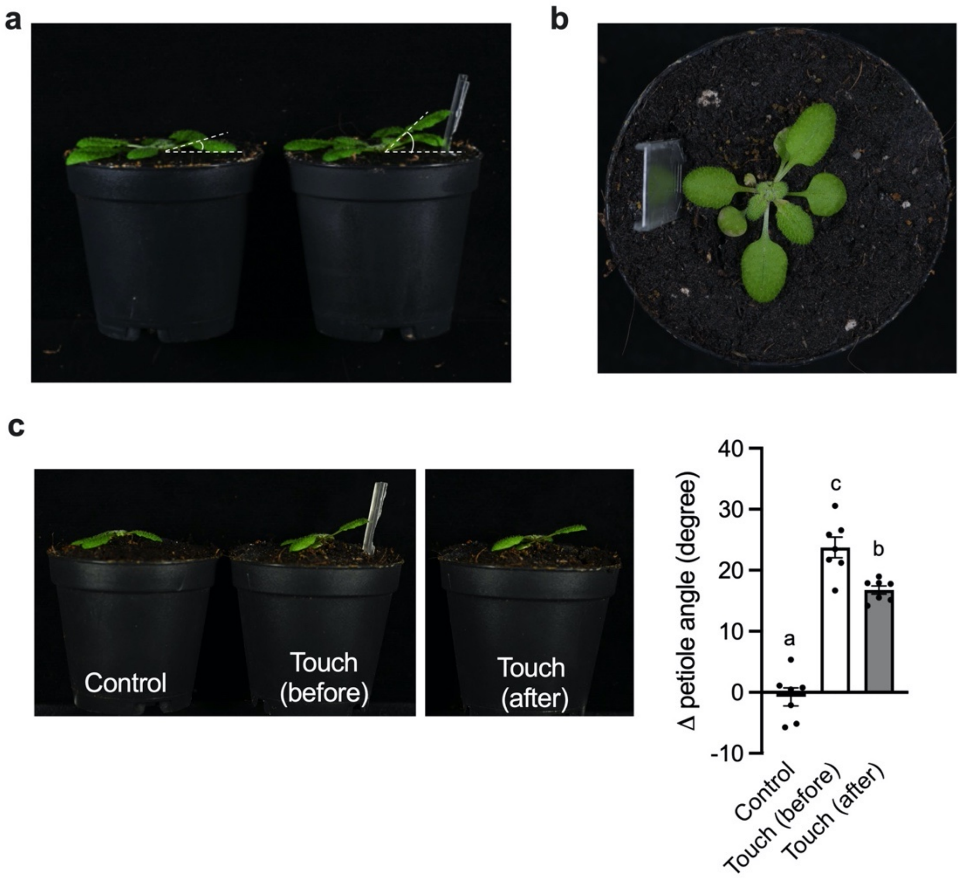

Supplementary Figure 13: Touch set-up with transparent tag. (**a**) Illustration of petiole angles (indicated with dotted white lines) in control and touch treatment. (**b**) Illustration of transparent tag next to the leaf tip trichome right before the touch experiment starts. (**c**) Illustration and differential petiole angle of control and touched leaf, after 24 h of touch treatment with tag still present [Touch (before)] and after removal of the tag [Touch(after)]. Data represent mean ± SE; n = 7 biologically independent replicates. Black dots represent the individual data. Different letters or asterisks indicate significant differences (one-way ANOVA with Tukey's post hoc test; $p < 0.05$).

Supplementary Table 1: Statistical analysis of Fig. 3**c**. P < 0.05 indicate statistically significant differences (Repeated measures ANOVA), while P > 0.05 indicate no statistically significant differences.

time	0 - 200 sec			200 - 500 sec		
	treatment	time	Treatment:Time	treatment	time	Treatment:Time
Position 1	p=0.8612	p<0.0001	P=0.8612	P=0.8707	p<0.0001	p<0.01
position 2	p=0.0992	p<0.0001	P=0.0001	p<0.01	p<0.0001	p<0.0001
position 3	p=0.5369	p<0.0001	P=0.0626	p=0.1590	p<0.0001	p<0.01
position 4	p=0.3702	p<0.0001	p<0.05	p=0.1276	p<0.0001	p<0.05
position 5	p=0.6977	p<0.0001	p<0.01	p=0.1660	p<0.0001	p=0.4737
position 6	p=0.8707	p<0.0001	p<0.01	p=0.5908	p<0.0001	p=0.2107

time	0 - 500 sec			500 - 1000 sec		
	treatment	time	Treatment:Time	treatment	time	Treatment:Time
Position 1	p=0.115	p<0.0001	p<0.0001	p<0.01	p<0.0001	p=0.0017
position 2	p<0.01	p<0.0001	p<0.0001	p=0.0005	p<0.0001	p=0.0064
position 3	p=0.2153	p<0.0001	p<0.0002	p=0.068	p<0.0001	p=0.1629
position 4	p=0.1952	p<0.0001	p<0.01	p=0.0693	p<0.0001	p<0.0001
position 5	p=0.3767	p<0.0001	p<0.01	p=0.0413	p<0.0001	p<0.0001
position 6	p=0.9171	p<0.0001	p=0.1435	p=0.0504	p<0.0001	p<0.0001

Supplementary Movie 1: Leaf tip touch $[Ca^{2+}]_{cyt}$ increase as reported with the cytosolic calcium biosensor *UBQ10p::GCaMP3*. The video is 85 x real time, time stamp indicates minutes and seconds of real time. Touch was induced by a transparent tag.

Supplementary Movie 2: Leaf tip touch-regulated $[Ca^{2+}]_{cyt}$ as reported with the cytosolic calcium biosensor *UBQ10p::GCaMP3* in the *glr3.1glr3.3glr3.6* mutant background. The video is 40 x real time, time stamp indicates minutes and seconds of real time. Touch was induced by a transparent tag.

Supplementary Movie 3: Touching trichomes at the leaf tip stimulates $[Ca^{2+}]_{cyt}$ as reported with the cytosolic calcium biosensor *UBQ10p::GCaMP3*. The video is 6 x real time, time stamp indicates minutes and seconds of real time. Touch was induced by a toothpick.

Supplementary Movie 4: Leaf tip touch $[Ca^{2+}]_{cyt}$ decrease as reported with the cytosolic calcium biosensor *UBQ10p::GCaMP3* in the *gl1* mutant background. The video is 40 x real time, time stamp indicates minutes and seconds of real time. Touch was induced by a transparent tag.

Supplementary Data

Supplementary Data 1: Differentially expressed genes (DEGs) in leaf tip and petiole bases upon touch treatment. Gene expression was determined in the leaf tip and the petiole base. Touch DEGs that are shared with those upon leaf tip FR treatment are identified separate from those that are specific to touch.

Supplementary Data 2: Gene IDs and Fisher exact values for comparisons in the different subsections of the VENN diagrams that show comparisons of gene expression in touch treatment with published mechanostimulation datasets.
