# Supplementary material for: Mechanodetection of neighbor plants elicits adaptive leaf movements through calcium dynamics
Source: Nat Commun. 2023 Sep 20;14:5827. doi: 10.1038/s41467-023-41530-0 (PMC10511701; doi:10.1038/s41467-023-41530-0)
Supplement: Supplementary file 3 — Description of Additional Supplementary Files [file 41467_2023_41530_MOESM3_ESM.pdf]

Description of Additional Supplementary Files

Supplementary Data Legends

Supplementary Data 1: Differentially expressed genes (DEGs) in leaf tip and petiole bases upon touch treatment. Gene expression was determined in the leaf tip and the petiole base. Touch DEGs that are shared with those upon leaf tip FR treatment are identified separate from those that are specific to touch.

Supplementary Data 2: Gene IDs and Fisher exact values for comparisons in the different subsections of the VENN diagrams that show comparisons of gene expression in touch treatment with published mechanostimulation datasets.

Supplementary Movie Legends

Supplementary Movie 1: Leaf tip touch $[Ca^{2+}]_{cyt}$ increase as reported with the cytosolic calcium biosensor *UBQ10p::GCaMP3*. The video is 85 x real time, time stamp indicates minutes and seconds of real time. Touch was induced by a transparent tag.

Supplementary Movie 2: Leaf tip touch-regulated $[Ca^{2+}]_{cyt}$ as reported with the cytosolic calcium biosensor *UBQ10p::GCaMP3* in the *glr3.1glr3.3glr3.6* mutant background. The video is 40 x real time, time stamp indicates minutes and seconds of real time. Touch was induced by a transparent tag.

Supplementary Movie 3: Touching trichomes at the leaf tip stimulates $[Ca^{2+}]_{cyt}$ as reported with the cytosolic calcium biosensor *UBQ10p::GCaMP3*. The video is 6 x real time, time stamp indicates minutes and seconds of real time. Touch was induced by a toothpick.

Supplementary Movie 4: Leaf tip touch $[Ca^{2+}]_{cyt}$ decrease as reported with the cytosolic calcium biosensor *UBQ10p::GCaMP3* in the *gl1* mutant background. The video is 40 x real time, time stamp indicates minutes and seconds of real time. Touch was induced by a transparent tag.
